# New treatment perspectives for negative symptoms

**DOI:** 10.1192/j.eurpsy.2024.73

**Published:** 2024-08-27

**Authors:** I. Bitter

**Affiliations:** Psychiatry and Psychotherapy, Semmelweis University, Budapest, Hungary

## Abstract

**Introduction:**

Persistent negative symptoms of schizophrenia are generally considered difficult to treat or treatment resistant. A large number of investigational drugs developed and or tested for the treatment of persistent negative symptoms failed to show efficacy leading to pessimism in treatment and disinvestment in treatment research of negative symptoms.

**Objectives:**

1. To demonstrate, that available treatment methods – both pharmacological and non-pharmacological - are in fact effective for the treatment of negative symptoms of schizophrenia 2. To shortly summarize new drug research in this field.

**Method:**

Review of research data.

**Results:**

The overall estimate for the placebo effect had a medium effect size, with a Cohen’s d value of 0.6444 (SE = 0.091).^1^ The estimates for the placebo effect were similar in the add-on and monotherapy studies. Amisulprid was superior to placebo, cariprazine was superior to risperidone, and “direct comparisons of antipsychotics in patients with predominant negative symptoms indicated no significant difference between amisulpride and olanzapine and between asenapine and olanzapine…”^2^_._ Various nonpharmacological interventions improved negative symptoms in randomized controlled trials relative to treatment as usual (e.g. social skills training, music therapy, non-invasive brain stimulation, mindfulness, and exercise-based interventions) ^3^ There is a progress in research with non-dopaminergic agents for the treatment of negative symptoms (e.g. pimavanserin, roluperidon, ulotaront).

**Conclusions:**

For medication classes other than antipsychotics and antidepressants, we found no reliable support for evidence-based recommendations for using these agents in the treatment of negative symptoms in clinical practice. Effect sizes for psychosocial interventions range from small to moderate. The use of placebo has shown a clinically significant positive effect on negative symptoms, a finding that warrants further research and provides a sense of optimism regarding potential therapeutic benefits.

1. Czobor, P., Kakuszi, B. and Bitter, I., 2022. Placebo response in trials of negative symptoms in schizophrenia: a critical reassessment of the evidence. Schizophrenia Bulletin, 48(6), pp.1228-1240. 2. Czobor, P., Bitter I. Pharmacologic treatment of negative symptoms: Focus on efficacy. In: Bitter I. (ed): Managing Negative Symptoms of Schizophrenia, Oxford University Press, 2020, p. 67
2. Savill, M. Psychosocial/non-pharmacologic treatment of negative symptoms: focus on efficacy. In: Bitter I. (ed): Managing Negative Symptoms of Schizophrenia, Oxford University Press, 2020, p. 87

**Disclosure of Interest:**

None Declared

